# A Revision of the Argentinean Endemic Genus *Eucranium* Brulléé (Coleoptera: Scarabaeidae: Scarabaeinae) with Description of one New Species and New Synonymies

**DOI:** 10.1673/031.010.20501

**Published:** 2010-12-07

**Authors:** Federico C. Ocampo

**Affiliations:** Laboratorio de Entomologíía, Instituto de Investigaciones de las Zonas ÁÁridas, CCT-CONICET Mendoza. CC 507, 5500. Mendoza, Argentina

**Keywords:** dung beetles, Eucraniini, Monte, taxonomy

## Abstract

The South American genus *Eucranium* Brulléé has been revised and now includes six species: *E. arachnoides* Brulléé, *E. belenae* Ocampo new species, *E. cyclosoma* Burmeister, *E. dentifrons* Guéérin-Mééneville, *E. planicolle* Burmeister, and *E. simplicifrons* Fairmaire. *Eucranium pulvinatum* Burmeister is a new junior synonym of *Eucranium arachnoides* Brulléé, and *Eucranium lepidum* Burmeister is a new junior synonym of *E. dentifrons* Guéérin-Mééneville. The following lectotypes and neotypes are designated: *Eucranium pulvinatum* Burmeister, lectotype; *Eucranium planicolle* Burmeister, lectotype; *Psammotrupes dentifrons* Guéérin-Mééneville, neotype; and *Eucranium lepidum* Burmeister, neotype. Description of the genus and new species, diagnosis and illustrations, and distribution maps are provided for all species. A key to the species of this genus is provided, and the biology and conservation status of the species are discussed.

## Introduction

The tribe Eucraniini (Scarabaeidae: Scarabaeinae) constitutes a monophyletic group and includes four genera, *Anomiopsoides* Blackwelder, *Ennearabdus* van Lansberge, *Eucranium* Brulléé, and *Glyphoderus* Westwood (Zunino 1983; Philips et al. 2002; Ocampo and Hawks 2006). This work showed that *Eucranium* consists of six species. The genus is endemic to Argentina and distributed in the Monte and Chacoan biogeographic provinces (based on Morrone 2006 schema); the previous records for Ecuador and Bolivia are erroneous (Martíínez 1959).

The genus *Eucranium* was originally described by Brulléé (1834) for one species, *E. arachnoides* Brulléé (1834). The name was originally proposed by Dejean (1833), but he did not properly describe the species. Later, Dejean (1836) cited the name *Eucranium arachnoides* in a catalog of Coleoptera of his collection. Westwood (1837) (nec Burmeister 1861) described the *Anomiopsis* genus as consisting of two species, *A. dioscorides* which was later synonymyzed with E. *arachnoides* Brulléé, and *A. sterquilinus* which was later transferred to the *Glyphoderus* genus (Westwood 1838). Guéérin-Mééneville (1838) described the *Psammotrupes* genus as consisting of one species, *P. dentifrons* Guéérin-Mééneville 1838 (= *E. dentifrons*). Laporte (1840) described *Pachysoma* (*Cyclodema*) *lacordairei* (= *E. arachnoides*) and indicated that this was the only species of *Pachysoma* in America. Blanchard (1841 pl. 10) described one species, *Anomiopsis aelianus* (= *E. arachnoides*). In 1845 Blanchard redescribed *A. aelianus* and referred to *E. arachnoides* and *A. dioscorides* with indication of their similarity to *A. aelianus.* Also Blanchard (1845) synonymized *Psammotrupes* with *Anomiopsis*, and placed *P. dentifrons* in the later genus. Blanchard (1845) proposed the synonymy of *Anomiopsis* with *Eucranium* (he assigned the name to Dejean). Lacordaire (1856) assigned the name *Eucranium* to Brulléé (1834) and provided a synonymy list [Anomiopsis Westwood, *Pachysoma* (*Cyclodema*) Laporte, and *Psammotrupes* Guéérin-Mééneville = *Eucranium* Brulléé]. Burmeister (1861) described three additional species of *Eucranium*: *E. cyclosoma*, *E. lepidum*, and *E. planicolle*; and redescribed *E. arachnoides.* In the same publication, Burmeister (1861), described *Anomiopsis* (nec *Anomiopsis* Westwood 1837) as a subgenus of *Eucranium* and placed four species in it [all these species are currently in the genus *Anomiopsoides* Blackwelder (1944) (Ocampo 2005)]. *Anomiopsis* Burmeister was elevated to generic level, and later the name was replaced with *Anomiopsoides* Blackwelder. Burmeister (1873) and Fairmaire (1893) each described one additional species of *Eucranium*: *E. pulvinatum* Burmeister and *E. simplicifrons* Fairmaire. The genus *Eucranium* was later listed in catalogs by Gillet (1911), Bruch (1911), Blackwelder (1944), and Martíínez (1959). The biology and behavior of *Eucranium* species were discussed by Zunino et al. (1989), Zunino (1991), Monteresino and Zunino (2003), Ocampo and Philips (2005), and Ocampo and Hawks (2006). The phylogenetic relationships of the genus were addressed by Zunino (1985), Philips et al. (2002), and Ocampo and Hawks (2006).

The other three Eucraniini genera, *Glyphoderus*, *Anomiopsoides,* and *Ennearabdus,* were revised by Ocampo (2004, 2005, 2007, 2010). The purpose of this contribution is to provide a taxonomic revision of the genus *Eucranium* including the description of one new species, to provide diagnosis and key to species, and to discuss their distribution, biology, and conservation status.

## Materials and Methods

Specimens were examined, dissected, and illustrated using a dissecting stereomicroscope (10–40x). Mouth parts and male genitalia were dissected and cleaned in a dilute solution (∼?10%) of potassium hydroxide and neutralized in a dilute solution (∼? 10%) of acetic acid. The male genitalia were placed in a glycerin-filled vial pinned under the specimen.

Body measurements, puncture density, puncture size, and density of setae were based on the following standards: Body length was measured from the middle of the anterior margin of the pronotum (at the middle) to the apex of the elytra, plus head length from the apex of clypeal process to the base of the head (head was measured separately because its variable position made it impractical to measure total body length). Body width was measured across mid-pronotum. Puncture density was considered ““dense”” if punctures were nearly confluent to less than 2 puncture diameters apart, ““moderately dense”” if punctures were 2–6 diameters apart, and ““sparse”” if punctures were separated by more than 6 diameters. Puncture size was defined as ““small”” if punctures were 0.02 mm or smaller, ““moderate”” if 0.02–0.07 mm, and ““large”” if 0.07 mm or larger. Setae were defined as ““sparse”” if there were few setae, ““moderately dense”” if the surface was visible but with many setae, and ““dense”” if the surface was not visible through the setae. Elytral carinae were counted from the elytral suture. Specimen labels were copied literally using ““/”” between lines and ““;”” between labels.

## Designation of neotypes and lectotypes.

Neotypes and Lectotypes were designated to provide the nomenclatural stability of the taxon studied, according to the Article 72 of the International Code of Zoological Nomenclature (1999).

Specimens for this research were collected or borrowed from and deposited in the following institutions and collections:

CMNC: Canadian Museum of Nature, Ottawa, Canada (R S Anderson, F. Géénier).
HECO: Hope Entomological Museum, Oxford, England (Mann D).
IAZA: Instituto Argentino de Investigaciones de las Zonas ÁÁridas, Mendoza, Argentina (F Ocampo).
IMLA: Fundacióón e Instituto Miguel Lillo, Universidad Nacional de Tucumáán, Tucumáán, Argentina (MV Colomo).
LEMQ: Lyman Entomological Museum, Mc Gill University, Quebec, Canada (S Boucher).
MACN: Museo Argentino de Ciencias Naturales, Buenos Aires, Argentina (A Roig).
MNHN: Musééum National d'Histoire Naturelle, Paris, France (O Montreuil).
MLPA: Museo de La Plata, La Plata, Argentina (A Lanteri).
UNSM: University of Nebraska State Museum, Lincoln, NE, USA (BC Ratcliffe).
USNM: United States National Museum, Washington D.C. USA (D Furth).

## Characters used and their taxonomic significance

Traditionally species in the *Eucranium* genus were described and recognized based mostly on the shape and length of the clypeal processes, and the pronotal and elytral sculptures. These characters rendered variable within species and were not reliable for species identification or description. In this work, new characters were explored and used to define species. Among these are Elytral pseudoepipleuron, pseudoepipleural angle with respect to elytral disc, elytral 8th striae (shape and sculpture), and shape and development of mesotibial spurs. Species male genitalia were studied in order to find species-specific patterns in the shape of the paremares, but the findings were not informative at this level. Internal sacs of paremeres were extracted and studied and these structures provided highly valuable information for phylogenetic analysis, however, they are impractical for species identification. Nevertheless, the information from the paremeres internal sacs is currently being used in a separate project on Eucraniini evolutionary biology (Ocampo et al in prep).

**Figure 1.**
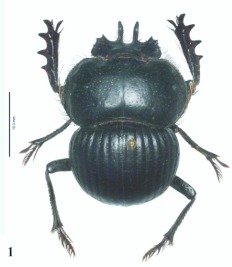
*Eucranium arachnoides*, male, dorsal view.

**Figure 2.**
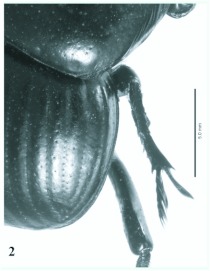
*Eucranium arachnoides*, elytron dorsolateral view. High quality figures are available online.

**Figure 3.**
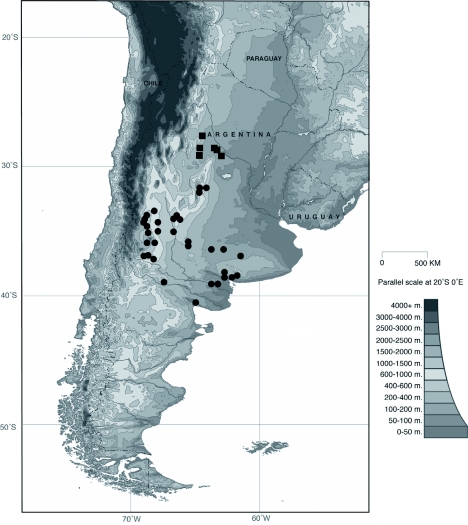
Distribution map of *E.* *arachnoides* (circles) and *E.* *simplicifrons* (squares). High quality figures are available online.

**Figure 4.**
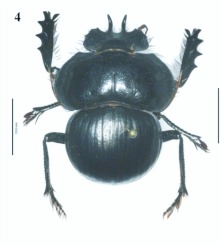
*Eucranium belenae*, male dorsal view.

**Figure 5.**
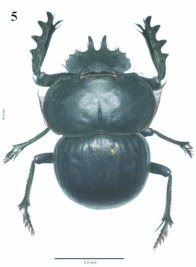
*Eucranium belenae*, female, dorsal view.

**Figure 6.**
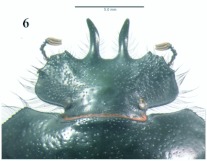
*Eucranium belenae*, male head, dorsal view.

**Figure 7.**
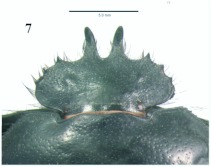
*Eucranium belenae*, female head, dorsal view. High quality figures are available online.

**Figure 8.**
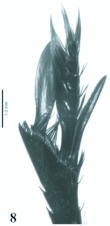
*Eucranium belenae*, tibial apex and mesotarsus.

**Figure 9.**
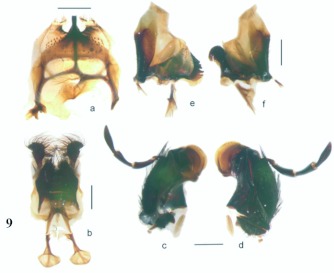
*Eucranium belenae*, mouthparts; 9A: labrum ventral view; 9B: labium ventral view; 9C, 9D: left maxilla, 9C: dorsal, 9D: ventral views; 9E: left mandible; 9F: right mandible (line scale = 1.0 mm).

**Figure 10.**
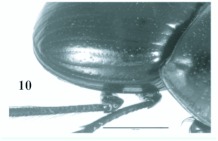
*Eucranium belenae*, elytron dorsolateral view.

**Figure 11.**
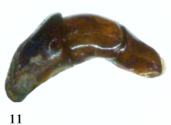
*Eucranium belenae*, male genitalia. High quality figures are available online.

**Figure 12.**
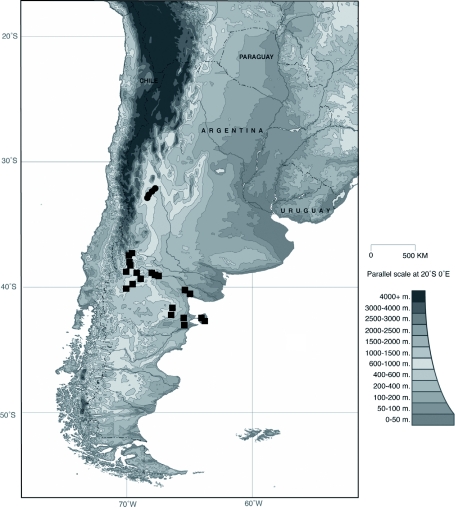
Distribution map of *E.* *belenae* (circles) and *E.* *dentifrons* (squares). High quality figures are available online.

**Figure 13.**
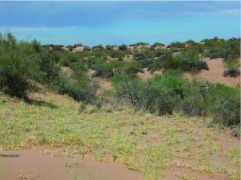
Habitat of *E.* *belenae* in Reserva Provincial Telteca, Mendoza, Argentina. High quality figures are available online.

**Figure 14.**
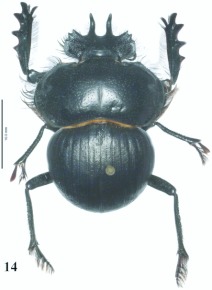
*Eucranium cyclosoma*, male dorsal view.

**Figure 15.**
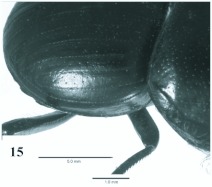
*Eucranium cyclosoma*, elytron dorsolateral view.

**Figure 16.**
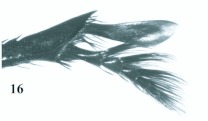
*Eucranium cyclosoma*, tibial apex and mesotarsus. High quality figures are available online.

**Figure 17.**
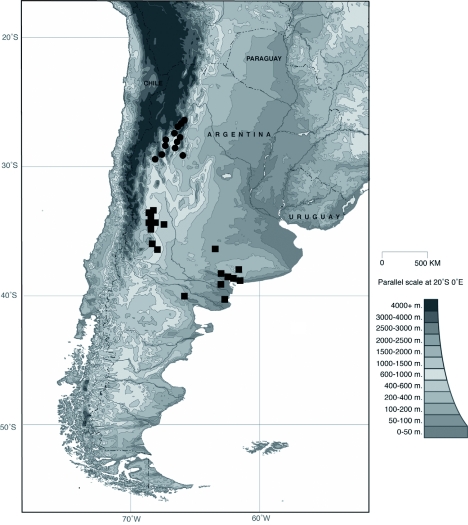
Distribution map of *E.* *cyclosoma* (circles) and *E.* *planicolle* (squares). High quality figures are available online.

**Figure 18.**
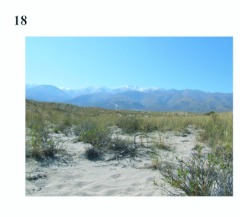
Habitat of *E.* *cyclosoma* close to Capillitas in Catamarca, Argentina.

**Figure 19.**
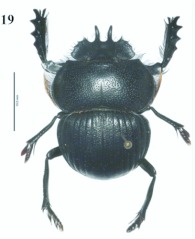
*Eucranium dentifrons*, male dorsal view.

**Figure 20.**
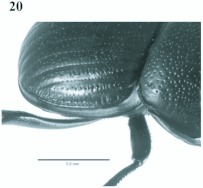
*Eucranium dentifrons*, elytron dorsolateral view.

**Figure 21.**
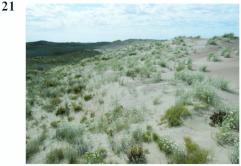
Habitat of *Eucranium dentifrons* in Peníínsula Valdes, Chubut, Argentina. High quality figures are available online.

**Figure 22.**
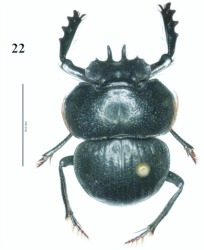
*Eucranium planicolle*, male dorsal view.

***Eucranium* Brulléé 1834 (Figures 1–26)**
*Eucranium* Brulléé 1834: 286.
  *Eucranium* Dejean 1833: 135, (*Nomen nudum*).
  *Eucranium* Dejean 1836: 150. *Anomiopsis* Westwood 1837: 13 (*nec* Burmeister 1861), subjective junior synonym. Type species *A. dioscorides* Westwood 1837: 13.
  *Anomiopsis* Westwood 1838: 159, *Psammotrupes* Guéérin-Mééneville 1838: 45, original description, subjective junior synonym. Type species *P. dentifrons* Guéérin-Mééneville 1838: 46.
  *Psammotrupes* Guéérin-Mééneville 1844: 74,.
  *Cyclodema* Laporte 1840: 68, (as subgenus of *Pachysoma* Mac Leay), junior synonym. Type species *Pachysoma lacordairei* Laporte 1840: 68.
  *Anomiopsis* Westwood 1838; Blanchard 1845: 225, synonymy list (= *Eucranium*).
  *Eucranium* Brulléé; Lacordaire 1856: 69.
  *Eucranium* Brulléé; Burmeister 1861: 58.
  *Eucranium* Brulléé; Burmeister 1873: 405.
  *Eucranium* Brulléé; Gillet 1911: 983.
  *Eucranium* Brulléé; Bruch 1911: 188.
  *Eucranium* Brulléé; Blackwelder 1944: 197.
  *Eucranium* Brulléé; Ocampo 2004: 2555.
Type species: *Eucranium arachnoides* Brulléé 1834, by monotypy.
**Diagnosis**
  The Eucraniini genus thacan be distinguished from other members of the New World Scarabaeinae by the following combination of characters: Body relatively large (13–30 mm), black (Figures 1, 4, 5, 14, 19, 22, 24); clypeus with two anterior processes well-developed (Figures 6, 7); pronotum without horns or tubercles; mesocoxae contiguous at the base; protarsi absent (male and female); mesotarsus shorter than metatarsus; and hind wings obsolete (flightless species).
**Redescription**
  Males and females. Body length 13.0–30.1 mm, width 9.6–19.18 mm. Color: head, pronotum and elytra dull to shiny black; venter dull to shiny black. *Head* (Figures 6, 7): Frons convex, surface smooth to punctate toward apex. Postocular lobes of parietal not depressed transversely. Cephalic carinae poorly developed or not developed. Eyes small, completely divided, dorsal and ventral halves not dorso-ventrally aligned; dorsal half slightly wider than ventral. Canthal area not developed, covered by gena. Gena well developed, genal posterior margin rounded. Clypeus transverse; surface rugose, punctate or rugo-punctate, punctures small to large. Clypeo-genal carinae present or obsolete. Clypeal anterolateral margin with three teeth, teeth well or poorly developed. Clypeal anterior margin with two well developed process, processes sexually dimorphic (females shorter and closer at base, well separated in males). Ventral surface with small punctures, ventral process well developed (narrow, not carina-like). Antennae 9-segmented, scape elbowed at base, antennomeres 2, 5, 6 slightly conical, 3, 4 elongate; antennal club longer than wide, lamellae with apex acute, surface tomentose except basal and central area of first lamella. *Pronotum* (Figures 1, 4, 5, 14, 19, 22, 24): Surface punctate, convex; strongly transverse, anterior margin sinuate, membrane not developed; antero-lateral and lateral margin broadly rounded, lateral portion bearing small irregular denticles, densely setose; setae recumbent, long; posterior angle broadly rounded; posterior margin slightly sinuate. All pronotal margins beaded, middle of anterior margin. Lateral pronotal fossae developed. *Elytra* (Figures 1, 4, 5, 14, 19, 22, 24): convex, globose, surface punctate; with 10 striae (including one adjacent to epipleuron). Epipleuron well-developed. *Hind wings:* obsolete (all brachypterous, flightless species). *Venter:* Surface smooth, glabrous or sparsely setose, prosternum pentagonal, anterior margins slightly concave. Mesosternum wider than long, mesometasternum suture visible or not. Metasternum flat, strongly narrowed in middle (metacoxae contiguous). Metepisternum 2.5–3 times longer than wide (at base). Ventrites narrower at middle. Pygidium with base grooved medially; disc slightly convex, sparsely punctate, punctures variable. *Legs* (Figures 1, 4, 5, 8, 14, 16, 19, 22, 24, 26): Protibia with 4 lateral teeth, dorsal surface with 4 well developed patches of setae, one at base (could be absent), one on base of teeth 2–3, one on apical surface on each side of tibial spur; protibial spur well developed curved. Protarsi not developed (males and females). Meso- and metafemorae longer then meso- and metatibiae respectively. Meso- and metatibiae long, slender, apex expanded; surface setose; setae long, slender. Mesotibial spurs developed, long; outer mesotibial spur slender or spatulalike. Meso- and metatibial externo-dorsal margin denticulate, each denticle bearing seta. Meso- and metatarsi well developed, becoming shorter from 1–5, densely setose, setae long; mesotarsi shorter than metatarsi. Meso- and metatarsal claws absent. *Male genitalia*: phallobase longer then parameres, symmetrical (Fig. 11).
  The genus name *Eucranium* is neutral in gender.
  The genus *Eucranium* consists in six known species.
**Distribution**
  ARGENTINA: Provinces of Tucumáán, Catamarca, Santiago del Estero, La Rioia Cóórdoba, San Juan, Mendoza, San Luis, La Pampa, Rio Negro, Neuquéén, and Chubut.
  Supplementary distribution maps, locality data, and modeled distribution of *E. arachnoides* are provided at: http://www.biofinity.unl.edu
**Phylogenetic relationships**
  Based on recently published phylogenetic analysis (Ocampo and Hawks, 2006, Monahan et al. 2007) and a more comprehensive analysis based on molecular and morphological data including all known species in the tribe (Ocampo et al unpublished) the genus *Eucranium* constitutes a monophyletic group. In these analysis, *Eucranium* is the sister taxon to the monotypic genus *Ennearabdus* and a clade composed by *Anomiopsoides* + *Glyphoderus.* Evidence indicates that *Eucranium* and *Ennearabdus* diverged early from the Eucraniini common ancestor, and *Anomiopsoides* and *Glyphoderus* diverged from a more recent ancestor (Ocampo and Hawks, 2006, Ocampo unpublished data).

***Eucranium arachnoides* Brulléé 1834 (Figures 1, 2, 3)**
*Eucranium arachnoides* Brulléé 1834: 289.
  *Anomiopsis dioscorides* Westwood 1837: 13, junior subjective synonym.
  *Pachysoma lacordairei* Laporte 1840: 68, junior synonym.
  *Anomiopsis aelinaus* Blanchard 1841: Fig 10.1 (1845), junior synonym.
  *Eucranium pulvinatum* Burmeister 1873: 405, **new synonym.**
**Type material**
  *Eucranium arachnoides* Brulléé Holotype female at MNHN labeled: ““*Eucranium* / *arachnoides* / dej. Tucuman.””; ““*Eucranium* / *arachnoides* / Brulléé / HOLOTYPE.””
*Anomiopsis dioscorides* Westwood, holotype female at HECO labeled: ““*Anomiopsis* / *dioscorides* West. / Trans Zool. Soc. pl 29.””; *E.* / *arachnoides* / Br. / J.J. E. Gillet det. / O.U.M.ix, 1910. / MS.by J.J.E.G.””; ““TYPE / WESTWOOD / Proc. Zool. Soc. 5.18.37 / p13 / Coll. Hope Oxon.””; ““TYPE Col: 429 / *Eucranium dioscorides* / West / HOPE DEPT. OXFORD””.
*Anomiopsis aelinaus* Blanchard, holotype male at MNHN labeled: ““Bai du San Blas””; ““MUSEUM PARIS / D'ORBIGNY 1834””; ““*Anomiopsis* / *aelianus* / Blanch””; ““TYPE””; ““*Anomiopsis* / *aelianus* Blanch / HOLOTYPE””.
*Eucranium pulvinatum* Burmeister lectotype male at MACN labeled: ““Cordo / va.””; ““Col. Antigua””; ““*pulvinatum* / COTYPUS / Burm.””; ““*Eucranium* / *pulvinatum* / Burmeister / 1973 / Syntypus””; ““*Eucranium* / *arachnoides* / Brulléé / A. Martíínez det. 1958””; ““*pulvinatum* 1””; ““*Eucranium* / *pulvinatum* / Burm. / LECTOTYPE / F. C. Ocampo desig. 2009””. **Lectotype here designated.** Two paralectotypes, one male and one female, at MACN labeled as lectotype except: ““pulvinatum 2”” and ““pulvinatum 3”” respectively. The type of *Pachysoma lacordairei* Laporte was not studied and it was nor possible to find it at MNHN where it should be deposited, this type is presumably lost.
**Diagnosis**
  Males (Figure 1) and females of *Eucranium arachnoides* can be distinguished from other *Eucranium* species by the following combination of characters: Elytron with pseudoepipleuron developed, pseudoepipleuron forming a <65°° angle with elytral disc (Figures 1, 2); elytron with outer margin of 8th striae not carinated, if carinated, carinae poorly defined and never reflexed (specimens from western and southern Mendoza province). Mesotarsus longer than mesotibial spur; body size: length 18.4–30.4 mm.
**Remarks**
  Based on morphological evidence it was concluded that there are no differences between *E. pulvinatum* Burmeister and *E. arachnoides* Brulléé and these species are placed in synonymy.
  *Eucranium arachnoides* is the species in the genus with the largest distributional range, and *E. arachnoides* presents considerable variation. Variation can be observed in the development of the pseudoepipleuron, body size, pronotal and elytral punctures, and male genitalia (slight differences in shape of parameres). These differences are not consistent among individuals of the same population. Based on the species concept used in this work to recognize *Eucranium* species, all these differences are attributed to intraspecific variation. Molecular information is needed to elucidate weather isolated populations (i.e., western Mendoza province, North Western Cóórdoba, and Eastern Buenos Aire constitute independent evolutionary lineages and if they should be treated as different species.
**Distribution**
  ARGENTINA (Figure 3). **Buenos Aires**: no more data (3); Argerich (1); Bahíía Blanca (4); Bahíía San Blas (1); Bajo Hondo (4); Carmen de Patagones (2); Estancia Barrau (6); Felipe Soláá (7); La Colina (1); Maza (3); Villa Iris (1). Cóórdoba: no data (6); ““Sur de Cóórdoba”” (1); San Javier (2); Las Rosas (3); Potrero de Góómez (1); Yacanto de San Javier (1). **La Pampa**: no more data (1); Gaviotas (1); Santa Rosa (1); Victorica (4). **Mendoza**: no more data (1); Agua Escondida (2); Aguada de los Ciegos (1); Arroyo el Rosario, Puesto las Gateadas (1); Arroyo La Rinconada (1); Base del Volcáán Diamante (1); Blanco Encalada (1); Caverna de los Tigres (1); Confluencia ríío Diamante and ríío Salado (1); Costa de Araujo (1); Dique Agua del Toro (4); Dique Agua del Toro (20 km S) (3); Dique El Carrizal (3); Divisadero (2); El Mollar (2); El Nihuil, Méédanos (1); Embalse El Nihuil (4); Fortíín Malargüüe (1); Huayqueríías (1); From RN 40 to Puesto Alvarado (2); Malargüüe (no more data) (4); Malargüüe, Los Corrales (5); Monte Comáán (1); ÑÑacuññáán (9); Pareditas (2); Reserva de la Bióósfera ÑÑacuññáán (11); Reserva Natural Laguna del Diamante (15 km SE) (19); Reserva Natural La Payunia, Puesto La Senillosa (1); Reserva Natural La Payunia, Los Relinchos (11); Reserva Natural La Payunia, Valle del Saino (1); RN 40 and Arroyo Yaucha (3); RN 40 (km 143) (1); RN 40 (S of Pareditas) (2); Road to Paso de Los Tigres (1); Salar del Nihuil (2); San Rafael (10). **Ríío Negro**: Coronel Góómez (2); Ríío Colorado (3). **San Luis**: no more data (1); Arizona (19); Balde (2); San Luis, Departamento Capital (7); El Volcáán (1); San Geróónimo (1). ““Patagonia”” no more data (1).
**Temporal distribution**
  January (79); February (12); March (12); April (5); May (1); July (2); August (4); September (7); October (4); November (15); December (51); no data (41).
**Biology and conservation**
  Biology and behavior of this species were recently discussed by Zunino et al. (1989), Monteresino and Zunino (2003), Ocampo and Philips (2005), and Ocampo and Hawks (2006). This species has the largest distributional range among *Eucranium* species. Populations of this species generally have a small, patchy distribution and consequently susceptibly to local extinction if changes in the environmental conditions occur. The only known populations of *E. arachnoides* that are currently in a protected area are those from Reserva Natural ÑÑacuññáán and Reserva Natural La Payunia in the Mendoza province.

***Eucranium belenae* Ocampo sp.n. (Figures 4–12)**
**Type material**
  *Eucranium belenae* Ocampo holotype male at IAZA labeled: ““ARGENTINA: Mendoza / R.N 142 km 107. N. Rva. / Telteca / 510m. 32°°15′?12″?S / 67°°49′?13″?W. 30/III/2009/ F. C. Ocampo””; ““*Eucranium* / *belenae* / HOLOTYPE / F. C. Ocampo””, Allotype female labeled as holotype except: ““*Eucranium* / *belenae* / ALLOTYPE / F. C. Ocampo.”” Twenty eight male and twenty female paratypes at IAZA: labeled as holotype. Eleven male and six female paratypes at IAZA labeled: ““ARGENTINA: Mendoza / Reserva Telteca. 32°°22≈?59.58″? S, / 68°°03′?14.16″? W. 548m. / 11-IV-2008. Col. L. Muññoz.”” Fourteen male and nine female paratypes at IAZA labeled as previous except: ““12-IV-2008””. Three male paratypes at IAZA labeled as previous except: ““13-IV-2008””. Three male paratypes at IAZA labeled as previous except: ““14-IV-2008””. One male paratype at IAZA labeled as previous except: ““15-IV-2008””. Three male and two female paratypes at IAZA labeled: ““ARGENTINA: Mendoza / R.N. 142 Km 107, N Rva. / Telteca. 510m. 32°°15′?12″?S / 67°°49′?13″?W. 1-III-2009. / KS Sheldon, FC Ocampo.”” Four male and eight female paratype at IAZA labeled as previous except: ““1/III/2009””. Four male and Four female paratype at IAZA labeled as previous except: ““FC Ocampo, K Sheldon”” and ““17/III/2009””. Seven male and three female paratypes at IAZA labeled: ““ARGENTINA: Mendoza / Lavalle. Telteca. 32°°22′?59.58″?S. / 68°°03′?14.16″?W. 548m. 05-II-2008. / Col. F. Ocampo, E. Ruiz, G. San Blas.”” One paratype at IAZA labeled: ““ARGENTINA: Mendoza / Lavalle. Telteca. 32°°22′?59.58″?S. / 68°°03′?14.16″?W. 548m. 27-XI-2007. / Col. F. Ocampo””. One male and one female paratypes at IAZA labeled: ““ARGENTINA: Lavalle / Puente Ríío Mendoza. 21 Feb. 2006. E. Ruiz.”” One male and one female paratypes at IAZA labeled: ““ARGENTINA: Mendoza / Reserva Telteca. 563m. S32°°23′?33″? W68°°03′?00″? / Jan-3-2002. F. C. Ocampo.”” Four female paratypes at IAZA labeled: ““R.A. Mza. Lavalle / Telteca / 3/2-14/3 /1995 / S. Roig / G. Flores.”” Four female paratypes at IAZA labeled: ““RA. Mza. Lavalle / Telteca /2/11 – 1/12 /1994 / G. Flores.”” One male and two female paratypes at IAZA labeled: ““Ra. Mendoza Tel- / teca 17/VIII-24/IX / 1996. Flores/Roig.”” One male and one female paratypes at IAZA labeled: ““Ra. Mendoza Tel- / teca 25/XI-25/XII / 1995. Flores/Roig.”” One male and one female paratypes at IAZA labeled: ““Ra. Mendoza Tel- / teca 25/IX-5/XI / 1996. Flores/Roig””. One male paratype at IAZA labeled: ““Ra. Mendoza Tel- / teca 14/8 -24/9/ / 1995 Flores/Roig.”” Two female paratype at IAZA labeled: ““Mendoza, Lavalle / Telteca 15-2 al 25-3-96. Col. G. Flores / IADIZA.”” One female paratype at IAZA labeled as previous except: ““15-4-95””. One male and three female paratypes at IAZA labeled: ““Ra. Mendoza Tel- / teca 3/II -14/III / 1995. Flores/Roig.”” One male and two female paratype at IAZA labeled: Mendoza, Lavalle / Telteca 1 al 15/12/ 94 Flores/Roig / IADIZA.”” One female paratype at IAZA labeled: ““Mendoza, Lavalle / Telteca 10/10 al 3/12/96 / Col. Gonzalez / IADIZA.”” Three male paratypes at IAZA labeled: ““RA. Mza. Lavalle / Telteca / 01.III.94 / G. Flores / IADIZA.”” One male paratype at IAZA labeled: ““Mendoza. Lavalle / Parque Telteca / 10.5.93 / M. Gonzáález / IADIZA”” One female paratype at IAZA labeled: ““Mendoza Lavalle Telteca 3-XII-96 / 6-I-97 Flores-Roig.”” One female paratype at IAZA labeled: ““Mendoza Lavalle Telteca 25/9-31/10 / 1995 Flores/Roig.”” One female paratype at IAZA labeled: ““Mendoza Lavalle Telteca 2/V-14/6 / 96 Flores/Roig / IADIZA.”” One male paratype at IAZA labeled: ““RA. Mza. Lavalle / El Encóón 12-IV-84 / IADIZA””; ““CE.000131 / IADIZA””. One male paratype at IAZA labeled: ““RA.Mza. Lavalle / Rva. Telteca / 15/X/03 / col. G. Debandi.”” One female paratype at IAZA labeled: ““RA. Mza. Lavalle / Telteca 01.III.94. / G. Flores / IADIZA.”” Three male and one female paratypes at IAZA with no data. All paratypes with a yellow paratype label: ““*Eucranium* / *belenae* / PARATYPE / F. C. Ocampo.””
**Type locality**
  Argentina, Mendoza, RN 142, km 107, 32°° 15′? 12″? S - 67°° 49′? 13″? W.
**Diagnosis**
  Males (Figures 4, 6) and females (Figures 5, 7) of *E. belenae* can be distinguished from other *Eucranium* species by the following combination of characters: Elytron with pseudoepipleuron not developed; elytral disc with interstriae becoming slightly convex toward margin, 8^th^ stria slightly sulcate; apex of mesotarsus reaching apex of outer mesotibial spur or not (viewed with tarsus extended parallel to tibial longitudinal axis) (Figure 8); outer mesotibial spur distinctively spatula-like, asymmetrical (Figure 8) (subject to wear); west-central Argentina.
**Description**
  Holotype male. Length 27.6 mm. Width 19.1 mm. Color black, surface shiny to matte. *Head* (Figures. 6, 7): Shape subrectangular, transverse. Frons slightly punctate. Frontoclypeal suture not evident, clypeogenal suture evident. Clypeogenal surface punctate, punctures slightly transverse. Genal posterior angle rounded; lateral margin smooth, setose. Clypeal surface obliquely angled downwards with respect to surface of frons; ventral surface developed between and on each side of clypeal medial processes, ventral process developed, acute. Clypeal medial process well developed, longer than clypeal length in middle, parallel; apex strongly reflexed; dorsal and external surface smooth; ventral half with fringe of setae. Clypeus with one tooth on each side, close to clypeogenal suture. Labrum (Figure. 9a) ventral surface, with medium brush not developed, replaced by sclerotized medial process; lateral files well developed setae thick; apical margin U-shaped strongly indented in middle with two convergent tips, lateral margins setose, setae continuous with apical fringe, slender; medial lobe of hypopharynx with transverse ridge of setae and spines. Labium ventral surface setose on anterior half and margin, setae black, long (Figure. 9b); anterior margin U-shaped, lateral margins oblique; labial palp with 3 palpomeres, palpomere 1 dilated, palpomeres 1–2 densely setose, palpomere 3 glabrous; glossal surface smooth, glabrous except apex of glossal flaps; lateral labial sclerites well developed, lateral arms of hypopharyngeal suspensorium longer than dorsal arm; oral arms not fused at middle, shorter than lateral arms. Maxillae (Figures 9c, 9d) articular process of cardo poorly expanded at apex, cardo external surface setose, setae long; stipital sclerite II surface sparsely setose, setae short, slender; stipital sclerites I, IV setose, setae long; stipital sclerite IV without medial longitudinal grove. Galea with articular sclerites well developed. Maxillary palpi with 4 palpomeres, palpomere 1, 2 subtriangular; 3, 4 subcilindrical; 4 1.5 times longer than 3. Mandibles (Figures 9e, 9f), molar lobe with serrate area on ventral half, well-developed; incisor lobe membranose surface setose at apex, setae minute; incisor lobe prostheca with lacking setae on basal half, few, short setae on apical half. *Pronotum* (Figures 4, 5): Surface punctate, punctures moderately dense to sparse, small to moderate in size. Lateral margin with long, dense setae on basal half, and moderately dense, short setae on apical half; margin beaded, denticulate on anterior half and at middle. *Elytron* (Figs 4, 5, 10): Striae slightly impressed, punctate; punctures small. Intervals sparsely punctate. Pseudoepipleura not developed, 8^th^ striae on sulcus (Figure 10). *Venter*: Metasternum sparsely punctate behind mesocoxae. *Legs* (Figures 4, 6, 8): Protibial teeth acute. Protibial spur with apex spatula-like, curved, acute. External mesotibial spur slightly curved, spatula-like asymmetrical on apical third, acute. Apex of mesotarsus reaches apex of outer mesotibial spur or not (viewed with tarsus extended parallel to tibial longitudinal axis). Male genitalia as in Figure 11.
**Allotype**
  Female (Figures 5, 7). Length 27.2 mm. Width 18.1 mm. As male except in the following respects: Clypeal medial process reflexed at apex, area between processes u-shaped; clypeal processes shorter than clypeal length in middle (Figure 7).
**Etymology**
  I take great pleasure in naming this species after my daughter Beléén Victoria.
**Remarks**
  Variation. Size: length 17.7–30.7 mm. Paratypes do not differ significantly from holotype. Variations are observed in puncture density and convexity of elytral intervals, been in some specimens more notorious than in primary types.
**Distribution**
  ARGENTINA (Figure 12). **Mendoza**: El Encóón (1); RN 142 Km 107 (114); RN 142 and Ríío Mendoza (2); Reserva Natural Telteca (27); Telteca (94).
**Temporal distribution**
  Jan (11); Feb (27); March (59); April (66); May (2); June (1); August (4); September (7); October (2); November (14); December (15).
**Biology and conservation**
  Specimens of *E. belenae* were observed carrying goat pellets and small pieces of dry horse dung at daylight hours (Video 1). Nocturnal activity also has been observed for this species, although no foraging has been noticed at night (Ocampo and Philips 2005). This species occurs in sand dunes in northeastern Mendoza province, which includes Reserva Natural Telteca; this protected habitat (∼?32,000 has) contributes to the conservation of *E. belenae* (Figure 13).

***Eucranium cyclosoma*Burmeister 1861 (Figures 14, 15, 16, 17)**
*Eucranium cyclosoma* Burmeister 1861: 60.
**Type material**
  *Eucranium cyclosoma Burmeister* holotype male at MNHN labeled: ““MUSEUM PARIS / Equateur””; ““*Eucranium* / *cyclosoma* / Burm. Dr. Dhorn / Ecuador””.
**Diagnosis**
  Males (Figure 14) and females of *E. cyclosoma* can be recognized distinguished from other *Eucranium* species by the following combination of characters: Elytron with pseudoepipleuron not developed (Figure 15); apex of mesotarsus reaches apex of outer mesotibial spur or not (viewed with tarsus extended parallel to tibial longitudinal axis) (Figure 16); mesotibial outer spur distinctively broad at apical 1/2, asymmetrical (Figure 16) (in some specimens this is character is not evident because the spur is worn down); elytral disc with interstriae smooth, evenly flat, 8^th^ stria not sulcate; northwestern Argentina; size 19.9–30.7 mm.
**Remarks**
  *Eucranium cyclosoma* is commonly mistaken for *E. arachnoides.* The original description of *E. cyclosoma* is based on a female specimen although the single specimen with corresponding type label is a male specimen. It is inferred that Burmeister made a mistake sexing the specimen and the specimen here considered the type is the specimen used by Burmeister to describe the species s. In his description Burmeister (1861) cites this species from Ecuador, but the genus has never been found there. Martíínez (1959) cites the species for Catamarca Tinogasta and mentioned the close resemblance of *E. cyclosoma* and *E. arachnoides.*
**Distribution**
  ARGENTINA (Figure 17). **Catamarca:** no more data (1); Andalgaláá (2); Barranca Larga (4); Beléén (3); Capillitas (3); Corral Quemado (3); El Arenal (1); RP 47 N of Capillitas (9); El Ingenio (1); Hualfíín (3); Isla de Sauce (1); Loma Negra (2); Pipanaco (1); Punta de Balasto, 12 Km W. Campo El Arenal (3); Punta de Balasto (S. of Santa Maria) (28); RN 40 KM 892 (3). **La Rioja**: no more data (4); Aimogasta (1); Aminga (1); Anillaco (6); Anillaco (2 km N) (12); RN 40 E of Guandacol (1); La Rioja (1). Salta: La Caldera, Campo Alegre (1). Tucumáán: Tafíí del Valle (1).
**Temporal distribution**
  January (21); February (52); March (7); November (23); December (4).
**Biology and conservation**
  Specimens of *E. cyclosoma* were observed carrying small pieces of dry horse dung at daylight hours over sand dunes in Catamarca province (Video 2, Figure 18). Conservation status of this species has not been assessed. This species in not known to occur in any protected area.

**Video 1.**
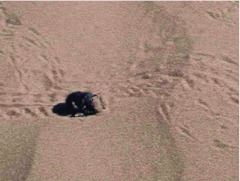
*Eucranium belenae* carrying a goat dung pellet over a sand dune in Telteca, Mendoza province, Argentina. .

***Eucranium dentifrons* (Guéérin-Mééneville 1838)(Figures 12, 19, 20, 21)**
*Eucranium dentifrons* (Guéérin-Mééneville 1838: 46) (*Psammotrupes*)
  *Psammotrupes dentifrons* Guéérin-Mééneville 1838:46.
  *Eucranium lepidum* Burmeister 1861: 61, **new synonymy.**
**Type material**
  *Psammotrupes dentifrons* Neotype male at IAZA labeled: ““ARGENTINA: Chubut / Pla. Valdes, Golfo Nuevo / Ea San Pablo. Méédanos / 42°° 42′? 28″? S 64°° 10′? 46″? W / 91 m. 1 Feb. 2006. 9:30-11:00 am. F.C. Ocampo, E. Ruiz, G. Salazar””; ““*Psammotrupes* / *dentifrons* / Guéérin-Mééneville / Neotype / F. C. Ocampo 2010.”” **Neotype here designated.**
  *Eucranium* / *lepidum* Burm. Lectotype at CMNC, labeled: ““ARGENTINA / Ríío Negro / San Antonio Oeste / R. N. Orfila leg. / Coll. Martíínez / Abr. 936.””; ““*Eucranium* / *lepidum* / Burm. / det J. Zidek 2000.””; ““*Eucranium* / *lepidum* / Burm. / Neotype / F. C. Ocampo 2010.”” **Neotype here designated.**
  The type material of *E. dentifrons* and *E. lepidum* could not be found despite the efforts to locate it in collections and it is presumably lost.
**Diagnosis**
  Males (Figure 19) and females of *E. dentifrons* can be distinguished from other *Eucranium* species by the following combination of characters: Elytron with well defined pseudoepipleuron, pseudoepipleuron forming a 45–60°° angle with elytral disc (Figures 19, 20); elytron with carina on outer margin of 8th stria, carina reflexed or rounded and reflexed; 7th interestria transversally rugose (most specimens), elytra with or without tubercles on humeral area; length 17.8–27.1 mm.
**Remarks**
  Based on morphological evidence it was concluded that there are no differences between *E. lepidum* Burmiester and *E. dentifrons* (Guéérin-Mééneville) and so these species are placed in synonymy.
  *Eucranium dentifrons* presents considerable variation in pronotal and elytral sculpture. Variation in puncture size and density on the pronotum and elytra is found among specimens of the same population and among populations. Variation is also found in rugosity on elytral interval seven, been in most specimens obvious and in some specimens slightly evident (although always present). These differences are more obvious among specimens from Neuquéén and western Rííio Negro province.
**Distribution**
  ARGENTINA (Figure 12). **Chubut**: no data (1); Gaiman (1); Puerto Madryn (CENPAT) (5); Telsen (2); Peníínsula Valdez, Estancia la Irma (5); Peníínsula Valdez, Estancia Los Méédanos (5); Peníínsula Valdez, Estancia San Pablo (23); Peníínsula Valdez (no more data) (8); Peníínsula Valdez, Playa Fracaso (1); Peníínsula Valdez, Puerto Piráámides (1); Peníínsula Valdez, Punta Delgada (5). **Neuquéén**: Aguada Florencia (2); Arroyo Picúún Leufúú (1); Colonia Centenario (1); Las Lajas (19 Km S) (4); Las Lajas, Cerro de la Cuchilla (4); Neuquéén (1); Picúún Leufúú (11); Piedra del ÁÁguila (6); Plaza Huincul (16); Ríío Agrio (N of Zapala) (7); RN 40 Km 2396, S of Las Lajas (14); RN 40, Bajada del Agrio (1); RN 40, El Marucho (1); Villa El Chocóón (2 km W) (3); Zapala (4). **Ríío Negro**: Barrancas del Gualicho (1); Cipolletti (1); Coronel Juan Joséé Góómez (2); General Roca (1); San Antonio Oeste, Las Grutas (8).
**Temporal distribution**
  January (38); February (62); March (16); April (8); September (1); October (6); November (2); December (12).
Biology and conservation
  Specimens of *E. dentifrons* have been observed caring and provisioning their borrows with guanaco dung pellets and small pieces of dry horse dung at daylight over sand dunes in Peníínsula Valdez, Chubut, and in Chocóón and near Las Lajas, Neuquéén (personal observation) (Figure 21).
  Conservation status of this species has not been assessed. The only protected area where *E. dentifrons* is known to occur is Peníínsula Valdééz in Chubut province.

**Video 2.**
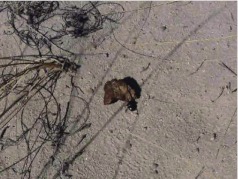
*Eucranium cyclosoma* carrying a piece of horse dung over a sand dune in Catamarca province, Argentina. .

**Figure 23.**
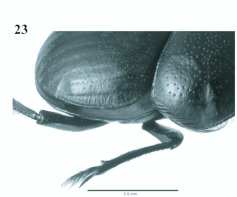
*Eucranium planicolle*, elytron dorsolateral view. High quality figures are available online.

***Eucranium planicolle*Burmeister 1861  (Figs. 17, 22, 23)**
*Eucranium planicolle* Burmeister 1861: 61.
**Type material**
  *Eucranium planicolle* Burmeister lectotype male at MACN labeled: ““Pampa / occid.””; ““*Eucranium* / *planicolle* / Burmeister / 1861””; ““Col. antigua””; ““*planicolle* 6””; ““*Eucranium* / *planicolle* / Burm. / LECTOTYPE / F. C. Ocampo det. 2009””. **Lectotype here designated**. One paralectotype male at MACN labeled as lactotype except: ““planicolle 5””.
**Diagnosis**
  Males (Figure 22) and females of *E. planicolle* can be distinguished from other *Eucranium* species by the following combination of characters: Elytron with well defined pseudoepipleuron, pseudoepipleuron forming an ∼?45–60°° angle with elytral disc (Figures 22, 23); elytron with outer margin 8th stria carinated, carina sharp and reflexed or rounded and reflexed (Figure 23); elytral 7th interestria smooth, never transversally rugose; elytra lacking small tubercles on humeral area; size relatively small, length 13.0–22.9 mm.
**Remarks**
  *Eucranium planicolle* is the smallest species in the genus and exhibits less variation than do other species of Eucranium.
**Distribution**
  ARGENTINA (Figure 17). No data (1). **Buenos Aires**: Argerich (1); Bahíía Blanca (3); Bahíía San Blas (1); Bajo Hondo (6); Coronel Pringles (1); Estacióón Delta near Monte Hermoso (13); Estancia Barrau (30 Km SW Villa Iris) (8); Monte Hermoso (3); Villa Iris (7). La Pampa: no more data (1); Anguil (1). Mendoza: no more data (6); 25 de Mayo (1); Agua Escondida (1); Dique Agua del Toro (2); Dique Agua del Toro (20 Km S.) (2); Monte Comáán (1); RP 143, Km 33 (1); Pareditas (3 Km S) (1); Pareditas (10 Km S) (1); Pareditas (22 Km S.) (1); Piedra Póómez (1); Reserva Natural Laguna del Diamante, 10 Km E. (1); RN 40, Puesto Alvarado (1); RN 40 and Arroyo Yaucha (1); RN 40, S of Pareditas (4); RP 150 (1). **Ríío Negro**: no more data (2); Ríío Colorado (8); RP 4 (60 KM N Valcheta) (2). **San Luis**: Departamento Capital (5).
**Temporal distribution**
  January (3); February (15); March (2); April (1); October (2); November (16); December (31).
**Biology and conservation**
  Specimens of *E. planicolle* are known to be diurnal and have been observed caring and provisioning their borrows with goat dung pellets in Mendoza province (Ruta Nacional 40 South of Pareditas) (personal observation). Conservation status of this species has not been assessed.

**Figure 24.**
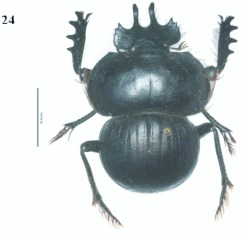
*Eucranium simplicifrons*, male dorsal view.

**Figure 25.**
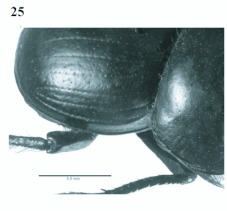
*Eucranium simplicifrons*, elytron dorsolateral view.

**Figure 26.**
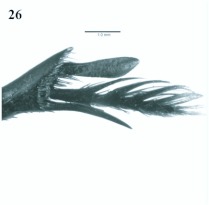
*Eucranium simplicifrons*, tibial apex and mesotarsus. High quality figures are available online.

***Eucranium simplicifrons* Fairmaire 1873  (Figures 3, 24, 25, 26)**
*Eucranium simplicifrons* Fairmaire 1873: 608.
**Diagnosis**
  Males (Figure 24) and females of *E. simplicifrons* can be distinguished from other *Eucranium* species by the following combination of characters: Elytron with or without pseudoepipleuron, if present, pseudoepipleuron forming an <65°° angle with elytral disc (Figures 24, 25); elytron with outer margin of 8th stria not carinated, if carinated, carina poorly defined and never reflexed; apex of mesotarsus when extended passes apex of outer mesotibial spur (viewed with tarsus extended parallel to tibial longitudinal axis) (Figure 26); mesotibial outer spur slightly broader on apical 1/3, nearly symmetrical (Figure 26); size, length 19.5–27.7 mm. Santiago del Estero.
**Remarks**
  *Eucranium simplicifrons* is the rarest species in the genus in entomological collections, presumably because it occurs in areas relatively poorly collected.
**Distribution**
  ARGENTINA (Figure 3). **Santiago del Estero**: Beltráán (2); Choya (8); El Charco (4); Fernáández (1); Guasayáán (1); Ramíírez de Velezco (1 Km N) (2).
**Temporal distribution**
  February (1); April (1); August (4); October (9); November (2).
**Biology and conservation**
  With the exception that the species is diurnal (personal observation) nothing is known about the biology of *E. simplicifrons.* Conservation status of this species has not been assessed; the species does not occur in any protected area.

**Key to species of *Eucranium* Brulléé**
1. Elytron with well defined pseudoepipleuron, pseudoepipleura forming a 45–60°° angle with elytral disc (Figures 20, 23); elytron with outer margin of 8^th^ striae carinated, carina sharp and reflexed or rounded and reflexed 2
1′?. Elytron with or without pseudoepipleuron, if present pseudoepipleuron forming a <65°° angle with elytral disc (Figures 2, 10, 15, 25); elytron with outer margin of 8^th^ stria not carinated, or if carinated, carinae poorly defined and never reflexed 3
2. Elytron with outer margin of 8^th^ stria carinated, carina sharp (Figures 22, 23); elytral 7^th^ interestria smooth, never transversally rugose; elytron lacking small tubercles on humeral area; size small (13.0–22.0 mm) *Eucranium planicolle* Burmeister (Figure 22)
2′?. Elytron with outer margin of 8th stria carinated, carina rounded; elytral 7^th^ interestria usually transversally rugose (Figures 19,20); elytron with or without tubercles on humeral area; size medium (17.8–27.1 mm) *Eucranium dentifrons* (Guéérin-Mééneville) (Figure 19)
3. Elytron with pseudoepipleuron absent 4
3′?. Elytron with pseudoepipleuron present, sometimes poorly developed *Eucranium arachnoides* Brulléé (Figure 1)
4. Mesotarsus as long as mesotibial spur or shorter (viewed with tarsus extended parallel to tibial longitudinal axis); outer mesotibial spur distinctively broad at apical 1/2, obviously asymmetrical (Figure 16) (spur subject to wear) 5
4′?. Mesotarsus when extended longer than mesotibial spur (viewed with tarsus extended parallel to tibial longitudinal axis); mesotibial outer spur slightly broad on apical 1/3, nearly symmetrical (Figure 26). Santiago del Estero *Eucranium simplicifrons* Lacordaire
5. Elytral disc with interstriae becoming slightly convex toward apical margin, 8^th^ stria slightly sulcate; west central Argentina (northeastern Mendoza province) *Eucranium belenae* Ocampo sp. nov. (Figures 4, 5)
5′?. Elytral disc with interstriae smooth, evenly flat, 8^th^ stria not sulcate. Northwestern Argentina (Catamarca, La Rioja) *Eucranium cyclosoma* Burmeister (Figure 14)

## Biogeography and Conservation

Morphological divergence of *Eucranium* and known geographic distribution suggest that the genus constitutes an endemic taxon in Chaco and Monte biogeographic provinces. Biogeographically, the Monte and Chaco are interesting regions forming an extensive transitional zone between Neotropical and Andean biotas (Rundel et al. 2007; Morrone 2006). South American deserts constitute very old habitats as elucidates from the presence of many endemic suprageneric and generic taxa well adapted to arid conditions (Roig Juññent et al. 2001; Ocampo and Hawks 2006, Ocampo et al. 2010). In the Monte and Chaco, endemic, relictual taxa coexist with other endemic taxa that would have speciated in the area but with sister groups in neighbouring non-desert regions (ex. Aclopinae, Allidiostomatinae (Scarbaeidae), *Taurocerastes* (Geotrupidae). Thus, the Monte and Chacoan biota have multiple origins with most genera being from Neotropical origin followed by groups with Patagonian or Andean affinities.

Species of *Eucranium* are distributed across a ∼?2000 km long (North-South) and 500 km wide (East West) range. Nevertheless, species in this genus show little sympatry, *E. arachnoides* and *E. planicolle* partially share they distributional range, while the rest of the species, *E. belenae*, *E. cyclosoma*, *E. dentifrons*, and *E. simplicifrons* are isolated from other species in the genus or only share a few localities (ex. *E. arachnoides* and *E. dentifrons* in Ríío Negro province). *Eucranium* species have high endemicity and populations have patchy distributions that make them susceptibly to local extinction if changes in the environmental conditions occur. Nothing is known for *Eucranium* species' population dynamics or habitat conservation status. Only two species, *E. belenae* and *E. dentifrons* are distributed within natural reserves or protected areas. It is well documented that there are genetic implications for small population size, among these it is a decline of genomic variation resulting from allelic loss (O'Brien 1994). According to Meffe *and* Carrol (1997) for the long term viability of a population it is important for it to maintain genetic variability which would enable the population to adaptively tolerate changes in environmental conditions. Further more, Keller et al. (2004), based on a study of a flightless ground beetle, provided evidence that even abundant species can be seriously affected by habitat fragmentation. Considering that all species in the genus *Eucranium* are flightless, and consequently with limited expansion or migration abilities, they are mostly associated to fragile environments (such as sand dunes), in order to preserve these species it is critical to understand their population dynamics and their habitat conservation status. *Eucranium* is characterized by its unusual morphology and unique biology and behavior, and it constitutes an old evolutionary lineage. Vane-Wright et al. (1991) proposed that these characteristics would make the genus *Eucranium* of high conservation value.
